# *In vivo* photoacoustic assessment of the oxygen saturation changes in the human radial artery: a preliminary study associated with age

**DOI:** 10.1117/1.JBO.26.3.036006

**Published:** 2021-03-23

**Authors:** Taehoon Bok, Eno Hysi, Michael C. Kolios

**Affiliations:** a Ryerson University, Faculty of Science, Department of Physics, Toronto, Canada; b Institute for Biomedical Engineering, Science and Technology, Toronto, Canada; c St. Michael’s Hospital, Keenan Research Centre for Biomedical Science, Division of Nephrology, Toronto, Canada

**Keywords:** photoacoustics, radial artery, red blood cell aggregation, oxygen saturation, age

## Abstract

**Significance:** We demonstrate the potential of probing the ${\mathrm{sO}}_{2}$ change under blood flow *in vivo* using photoacoustic (PA) imaging and sheds light on the complex relationship between RBC aggregation and oxygen delivery.

**Aim:** To conduct *in vivo* assessments of the ${\mathrm{sO}}_{2}$ in the radial artery of healthy volunteers and simultaneously probe the relation between the ${\mathrm{sO}}_{2}$ and hemodynamic behavior such as red blood cell (RBC) aggregation.

**Approach:** The effects of PA-based measurements of blood hemodynamics were studied as a function of the subjects’ age (20s, 30s, and 40s). The pulsatile blood flow in the human radial artery of 12 healthy subjects was imaged in the 700 to 900 nm optical wavelength range using a linear array-based PA system.

**Results:** The PA power when blood velocity is minimum ($Pa_{\max}$) was larger than the one attained at maximum blood velocity ($Pa_{\min}$), consistent with predictions based on the cyclical variation of RBC aggregation during pulsatile flow. The difference between $Pa_{\min}$ and $Pa_{\max}$ at 800 nm ($\Delta Pa_{800}$) increased with age (1.7, 2.2, and 2.6 dB for age group of 20s, 30s, and 40s, respectively). The ${\mathrm{sO}}_{2}$ computed from $Pa_{\max}$ was larger than the one from $Pa_{\min}$.

**Conclusions:** The $\Delta Pa_{800}$ increased with participant age. The $\Delta Pa_{800}$ metric could be a surrogate of noninvasively monitoring the age-induced changes in RBC aggregation. The ${\mathrm{sO}}_{2}$ change during a cycle of pulsatile blood flow also increased with age, demonstrating that RBC aggregation can affect the ${\mathrm{sO}}_{2}$ change.

## 1 Introduction

Red blood cells (RBCs) play an important, physiologically significant role in the human body, affecting hemodynamics as well as governing an oxygen transport. The oxygen-carrying capacity of RBCs is driven by the partial pressure of oxygen in blood vessels and the tissues they supply and is systemically assessed through metrics of oxygen saturation (${\mathrm{sO}}_{2}$).^1^ The hemodynamic aspects of RBCs, namely their flow profiles, pressures, and velocities, across the cardiovascular system play an equally important physiological role.^2^ Pathophysiological changes in the hemodynamic behavior of RBCs affect a wide variety of disorders.^3^ A phenomenon that is affected by the hemodynamic behaviors of RBCs is their aggregability, defined as the ability of the cells to form rouleaux in the presence of plasma proteins.^4^ This naturally occurring phenomenon is attributed to the cyclical variation of the shear forces forming when blood velocity gradients change during flow.^5^ It is thus affected by the hemodynamic behavior of vessels^6^ as well as RBC membrane factors that impact the electrostatic and steric interactions between cells.^7^ The clinical significance of RBC aggregation becomes apparent in conditions where hyperaggregability has been reported. These include myocardial infarctions, bacterial infections, type 2 diabetes, and sickle cell disease.^5^ The study of RBC aggregation as a hemodynamic phenomenon has provided invaluable insights into the biophysical properties of this process.^8^^,^^9^ However, measurements of the ${\mathrm{sO}}_{2}$ in the presence of RBC aggregation have not been possible, impeding the assessment of the significance aggregation to the process of oxygen delivery.

The ${\mathrm{sO}}_{2}$ is universally recognized as one of the vital signs in medicine along with pulse rate, respiratory rate, temperature, and blood pressure.^10^^,^^11^ This metric measures the percentage of hemoglobin binding sites that are bound with oxygen in RBCs in the blood. Oxygen metabolism is tightly regulated in the body because hypoxemia (abnormally low blood oxygen level) can lead to acute adverse effects in several organ systems.^10^^,^^12^^–^^15^ Recently, the decreased ${\mathrm{sO}}_{2}$ of COVID-19 patients measured at hospital admission was reported as one of the strongest predictors of critical illness, with silent hypoxia concurrently occurring with other symptoms such as shortness of breath.^16^^–^^18^

In general, clinical ${\mathrm{sO}}_{2}$ measurements are conducted using a pulse oximeter that attaches at the tip of the patient’s finger and is based on optical spectral differences between oxyhemoglobin (HbO) and deoxyhemoglobin (HbD). Pulse oximeters produce a measure of oxygenation which is sensitive only to the arterial vessels in the finger, not the veins. Another relevant clinical measurement is the arterial ${\mathrm{sO}}_{2}$, or “${\mathrm{SaO}}_{2}$,” a metric that is assessed using invasive blood draws from the radial artery followed by co-oximetry or blood gas analysis or indwelling catheter probes.^19^ However, in cases when peripheral perfusion is poor, as in states of hypovolemia, hypothermia, vasoconstriction, low cardiac output, and low mean arterial pressure, pulse oximeter readings become unreliable.^20^ Moreover, a variety of other clinical conditions such as vaso-occlusive crises in sickle cell anemia, severe anemia, methemoglobinemia, sepsis, or septic shock can influence the accuracy of pulse oximeter readings.^19^ Imaging techniques such as blood oxygen level-dependent magnetic resonance imaging (BOLD MRI),^21^ positron emission tomography (PET),^22^ or diffuse optical tomography or optical spectroscopy^23^^,^^24^ can assess the tissue oxygenation *in vivo*. Still, their millimeter-scale spatial resolutions limit their ability to assess the blood ${\mathrm{sO}}_{2}$ directly. In particular, BOLD MRI signals measure only HbD changes,^25^ which are likely independent of the blood ${\mathrm{sO}}_{2}$ while PET uses ionizing radiation. Furthermore, these techniques are not portable and cannot be used to monitor ${\mathrm{sO}}_{2}$ in a clinical setting or in a point of care setting. For the noninvasive measurement of ${\mathrm{sO}}_{2}$ in more centrally located vessels, near-infrared spectroscopy (NIRS)-based oximetry is widely used.^26^ However, NIRS-based tissue oximeter cannot be used in a simultaneous assessment of the relationship between the ${\mathrm{sO}}_{2}$ and blood dynamics.

Photoacoustic (PA) imaging is an emerging technique in biomedical optics. It can provide not only anatomical structure but also functional information, complementing conventional ultrasound (US) imaging. Also, it provides greater penetration depth than conventional optical imaging methods since it relies on the detection of acoustic waves rather than ballistic photons.^27^^,^^28^ The high absorbance of hemoglobin within RBCs and the oxygen-dependent absorption of hemoglobin allows PA imaging to probe the microvasculature ${\mathrm{sO}}_{2}$, *in vitro*, as well as *in vivo*.^29^^–^^31^ PA imaging is capable of noninvasive imaging of blood flow as well as simultaneous assessment of ${\mathrm{sO}}_{2}$, being utilized as the state-of-the-art tool for clinical applications such as investigation on the correlation between tissue characterization and oximetry.

However, no PA ${\mathrm{sO}}_{2}$ studies have ever examined the impact of blood’s hemodynamic behavior in the estimation of this important parameter. There are several methods to assess RBC aggregation, such as erythrocyte sedimentation rate, centrifugation methods, microscopic methods, low shear viscometry, US imaging, and analysis of light transmission (or reflection) of RBC suspension.^32^ The noninvasive measurement of RBC aggregation has been widely investigated using US imaging.^33^^–^^35^ RBC aggregation has also been used as a clinical biomarker for inflammation monitoring among other pathologies.^8^^,^^9^^,^^36^ However, US imaging probes the structure of RBC aggregates and does not directly measure physiological function of RBCs. As such, the assessment of both ${\mathrm{sO}}_{2}$ and hemodynamic behavior under blood flow needs to be independently assessed using noninvasive biomedical imaging using conventional optics^37^^–^^40^ and US,^33^^–^^35^ respectively, but not in the same setting. Our group has reported on *in vitro* measurements that show the correlation between the ${\mathrm{sO}}_{2}$ and RBC aggregation using PA spectroscopy in static and flow conditions.^41^^–^^43^ Through carefully controlled *in vitro* conditions, we demonstrated that during a pulsatile cycle in a simulated pulsatile blood flow, the PA amplitude increased with decreasing flow velocity and decreased with increasing velocity. These changes in PA amplitude were due to RBC aggregation during small velocities and disaggregation during increased velocities and consistent with the findings widely reported using conventional US methodology.^33^^,^^35^ In addition to RBC aggregation, the PA-based ${\mathrm{sO}}_{2}$ also cyclically changed in-phase with RBC aggregation. These results suggest that RBC aggregates inhibited $O_{2}$ release. In this paper, we describe the first *in vivo* evidence for the RBC aggregation-induced alterations in the ${\mathrm{sO}}_{2}$ measured in the radial artery of healthy subjects. Furthermore, a complex age-dependent correlation between the ${\mathrm{sO}}_{2}$ and RBC is discussed.

## 2 Materials and Methods

### 2.1 Subject Recruitment and PA Imaging Protocol

The human subject study was approved by the Research Ethic Board of Ryerson University (REB 2017-040). Healthy subjects were recruited based on the inclusion criteria listed in Table 1.

**Table 1:** Subject criteria and information.

Eligibility criteria	– Healthy adult without any history of ineligibility criteria		
	– Ages 20 to 69		
Ineligibility criteria	– Self-reported history of		
	• Blood disorders		
	• Diabetes		
	• High blood pressure		
	• Poor circulation in the brain, neck, or legs		
	– Pregnant women		
Group	20s	30s	40s
N	6	3	3
Age	24.5±2.8	35.3±3.2	41.7±1.5
BMI	23.7±2.8	24.3±1.6	28.3±4.0

Note: N: number of subjects, BMI: body mass index ($\mathrm{kg}/m^{2}$).

**Fig. 1:**
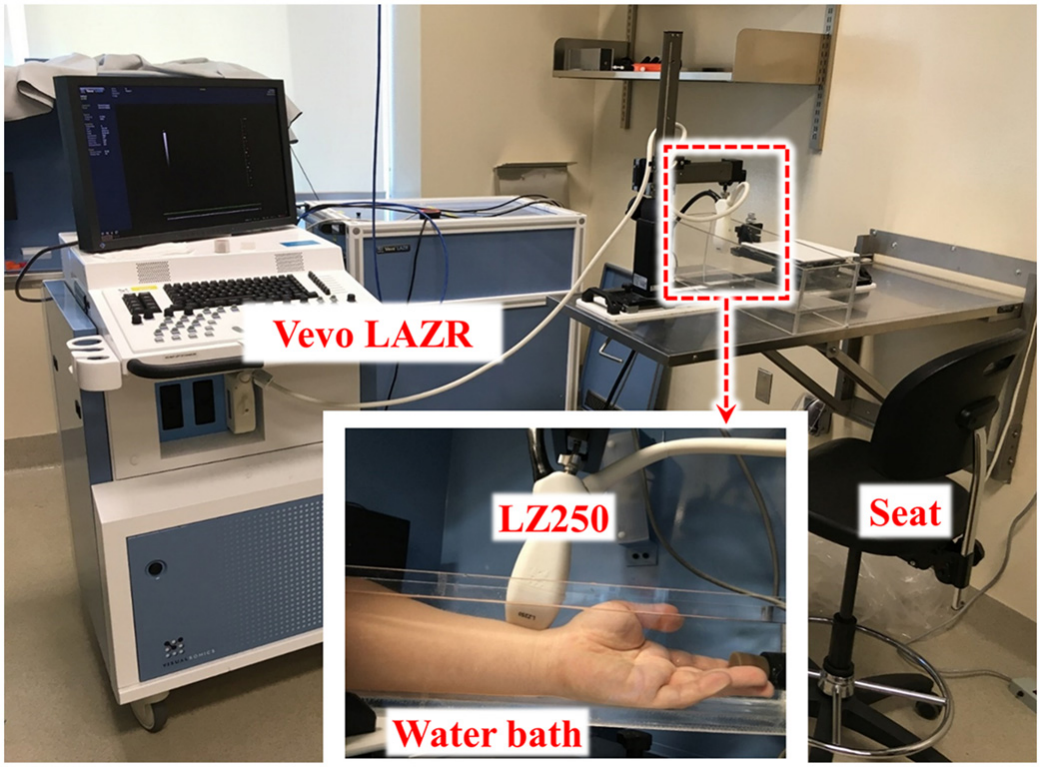
Photograph of PA imaging system and a PA probe positioned over the region of the interest close to the wrist of a subject.

The subject sat down on a chair and immersed their left arm in a degassed warm (36°C) water bath (Fig. 1). PA imaging was performed with a linear-array probe equipped PA imaging system (Vevo LAZR; LZ250-21 MHz of central frequency, 13 to 24 MHz of bandwidth and 256 elements, FUJIFILM Visualsonics, Toronto, Ontario, Canada)^44^ at the near-infrared wavelength (700, 750, 800, 850, and 900 nm). The pulsed-wave Doppler velocity (V) was measured to locate the radial artery, and the measurement system was then switched to the PA imaging mode. A PA B-mode image was acquired to measure the pulsatile blood flow in the radial artery for 10 s at each optical wavelength (λ). The total time that subjects’ arms were immersed in water was <5  min. The time trace was acquired for each single wavelength then time-shifted to create a combined multispectral dataset in postprocessing. The detailed methods were described in our previous study.^42^^,^^45^

### 2.2 Data Acquisition and Postprocessing: *V*, *Pa*, and sO_2_

**Fig. 2:**
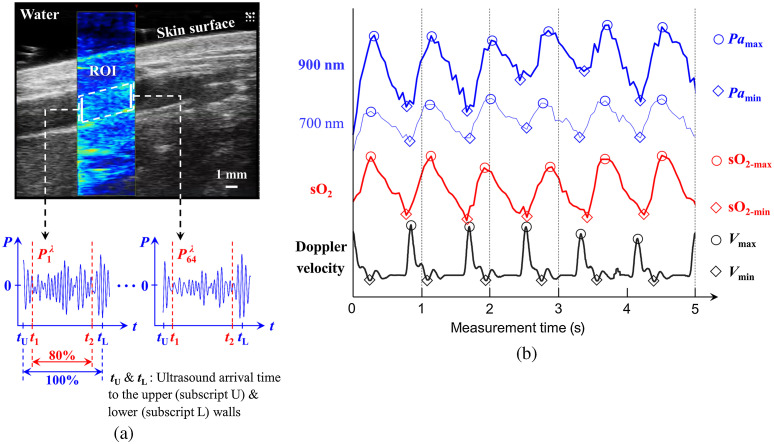
(a) Representative US (gray scale) and PA, PA (color scale) image of the radial artery. The PA RF power (Pa) from the ROI was computed by taking an average of the root-mean-square of each RF signal for each optical wavelength (λ). (b) Representatives of Pa (900-nm-thick blue and 700-nm-thin blue), oxygen saturation (${\mathrm{sO}}_{2}$, red), and the Doppler velocity (V, black). The subscripts max and min represent the maximum (denoted as a circle) and minimum (denoted as a diamond) value for each parameter.

A representative coregistered US (gray scale) and PA (color scale) image of the radial artery was shown in Fig. 2(a). The second quarter of the full field of view was chosen as a region of interest (ROI) to maximize the PA image acquisition rate to 20 Hz. To avoid boundary-buildup or edge detection artifact from the upper and lower vessel walls, the upper and lower boundaries of the ROI were chosen at 10% margin within the vessel lumen. In the coregistered US image, the upper and lower vessel walls were tracked at each frame. For each wavelength and each subject, 200 frames of US images (20  frame/s) were analyzed to track the vessel wall. From the upper and lower walls tracked, the upper and lower boundaries for the ROI were computed by 10% margin within the vessel lumen. For example, if the locations of two walls were at 1 and 2 mm in depth (corresponding to $t_{U}$ and $t_{L}$, respectively), the locations of two boundaries of ROI were 1.1 and 1.9 mm in depth (corresponding to $t_{1}$ and $t_{2}$, respectively) as shown in Fig. 2(a). The horizontal width of the ROI was fixed as a second quarter of the full field of view, whereas the vertical length (related to the vessel diameter) of the ROI was dependent on each frame. The VevoLAZR PA imaging system provides access to the prebeamformed RF data for all 256 transducer elements. These data are then beamformed postacquisition, and the amplitude of each signal is used to reconstruct the PA images shown in Fig. 2(a). The ROI is then selected from this reconstructed PA image. From beamformed radiofrequency (RF) PA signals (64 out of 256 elements) in the ROI, the PA power (Pa) was computed by taking an average of the root-mean-square of each RF signal for each λ, and addressed by

$$Pa(\lambda )=\frac{1}{64}\overset{64}{\underset{n=1}{\sum }}\frac{1}{E(\lambda )}\sqrt{\frac{1}{\Delta t}\int_{t_{1}}^{t_{2}}{\left|P_{n}^{\lambda }(t)\right|}^{2}dt},$$ (1)

where E is the laser energy in J, which measured by Vevo LAZR, $\Delta t=t_{2}-t_{1}$, $t_{1}$ and $t_{2}$ represent the time in the RF signal, corresponding to the upper and lower boundaries of the ROI, respectively, as shown in Fig. 2(a), P is PA amplitude in voltage, n is the element number of US detector. The velocity (V) of the pulsatile blood flow measured for 10 s. The number of pulsatile cycles was dependent on the subject (ranging from 11 to 15 cycles during the 10-s measurement for all subjects). The ${\mathrm{sO}}_{2}$ was estimated using the optical absorption at two applied wavelengths, which was derived from^46^

$$\mu_{a}(\lambda )=\varepsilon_{\mathrm{HbO}}(\lambda )[\mathrm{HbO}]+\varepsilon_{\mathrm{HbD}}(\lambda )[\mathrm{HbD}],$$ (2)

$${\mathrm{sO}}_{2}=\frac{\left[\mathrm{HbO}\right]}{\left[\mathrm{HbO}]+[\mathrm{HbD}\right]}=\frac{\mu_{a}(\lambda_{2})\varepsilon_{\mathrm{HbD}}(\lambda_{1})-\mu_{a}(\lambda_{1})\varepsilon_{\mathrm{HbD}}(\lambda_{2})}{\mu_{a}(\lambda_{1})\Delta \varepsilon_{\mathrm{Hb}}(\lambda_{2})-\mu_{a}(\lambda_{2})\Delta \varepsilon_{\mathrm{Hb}}(\lambda_{1})},$$ (3)

where $\mu_{a}$ is the absorption coefficient in /cm; $\varepsilon_{\mathrm{HbO}}$ and $\varepsilon_{\mathrm{HbD}}$ are the known molar extinction coefficients of oxygenated hemoglobin (HbO) and deoxygenated hemoglobin (HbD), respectively, in /cm/M; $\Delta \varepsilon_{\mathrm{Hb}}=\varepsilon_{\mathrm{HbO}}-\varepsilon_{\mathrm{HbD}}$; [HbO] and [HbD] are the molar concentrations of HbO and HbD, respectively, in M; $\lambda_{1}$ and $\lambda_{2}$ are two different λ (700 and 900 nm in this study). P can hence substitute for $\mu_{a}$. P measured at 700 and 900 nm were synchronized to calculate the ${\mathrm{sO}}_{2}$ using Eq. (2), as shown in Fig. 2(b). P was acquired at each of the wavelengths for 10 s, and then the wavelength was switched to the next one. The time difference between acquisition at wavelengths was 20 s, which is 10 s for wavelength switching and 10 s for acquisition at the wavelength. The ${\mathrm{sO}}_{2}$ assessment using our technique has been validated in previous studies^47^ and independently validated in pre- or clinical studies.^48^ Since the position and geometry of illuminated area were the same, the fluence effect could be minimized. Also, the effect of wavelength on fluence was somewhat compensated by using normalization to the energy at each wavelength. The representatives of Pa, oxygen saturation (${\mathrm{sO}}_{2}$, red), and the Doppler velocity (V, black) were shown in Fig. 2(b).

The minimum ($Pa_{\min}$ and ${\mathrm{sO}}_{2\text{-}\min}$) and maximum ($Pa_{\max}$ and ${\mathrm{sO}}_{2\text{-}\max}$) values of Pa and ${\mathrm{sO}}_{2}$ were, respectively, averaged for 10 s to compare the variation in Pa and ${\mathrm{sO}}_{2}$ during the pulsatile blood flow as a function of λ and age. Two sample t-test was conducted using built-in MALTAB function “ttest2.m,” in terms of the ${\mathrm{sO}}_{2}$ and Pa changes versus age group.

## 3 Results

### 3.1 PA Power versus Optical Wavelength

**Fig. 3:**
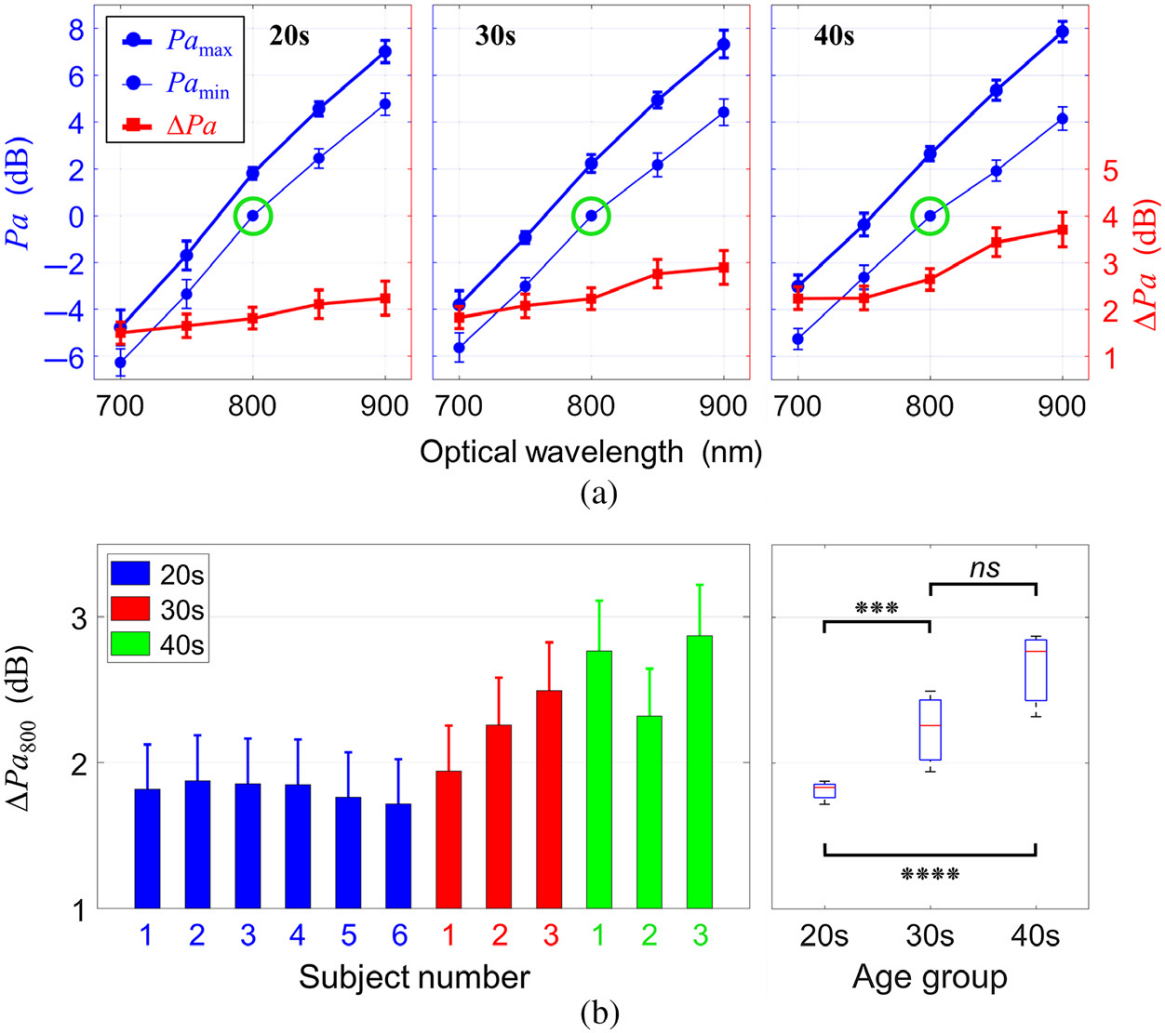
(a) PA power (Pa) as a function of optical wavelength for each group of age 20s, 30s, and 40s. The thick and thin blue lines represent $Pa_{\max}$ and $Pa_{\min}$, respectively. The thick red line represents the variation from $Pa_{\min}$ to $Pa_{\max}$ (ΔPa). The green circle indicates the isosbestic point (HbO and HbD have the same optical absorption). The error bars represent the standard deviation from the number of cycles of the pulsatile flow for all subjects, i.e., 75, 39, and 38 cycles for each age group, respectively. (b) The Pa difference between $Pa_{\min}$ and $Pa_{\max}$ at an isosbestic point 800 nm ($\Delta Pa_{800}$) for all subjects and the corresponding box and whisker plot (based on the average value of $\Delta Pa_{800}$ for each subject) for each age group. The error bars represent the standard deviation from the number of cycles of the pulsatile flow for each subject. ns: not significant, *** : p≤0.001, **** : p≤0.0001.

The PA magnitude was dependent on the optical λ as well as the study subjects. To compare this parameter, the $Pa_{\max}$ and $Pa_{\min}$ for all λ were normalized to the $Pa_{\min}$ at an isosbestic point 800 nm (the point at which HbO and HbD have the same optical absorption) for each subject. The Pa dependence on the λ for each subject age group is shown in Fig. 3(a). The $Pa_{\min}$ at 800 nm was set to 0 dB as a reference [green circles in Fig. 3(a)]. Both $Pa_{\max}$ and $Pa_{\min}$ increased with λ for all groups, as shown in Fig. 3(a). In addition, the difference between $Pa_{\min}$ and $Pa_{\max}$ (ΔPa) increased with the λ. The average values of $Pa_{\max}$, $Pa_{\min}$, and ΔPa at 700, 800, and 900 nm for all groups were tabulated (Table 2). The Pa difference between $Pa_{\min}$ and $Pa_{\max}$ at 800 nm ($\Delta Pa_{800}$) for all subjects is shown in Fig. 3(b)-left. The error bars represent the standard deviation from the number of cycles of the pulsatile blood flow for each subject. The box and whisker plot based on the average values of $\Delta Pa_{800}$ of each subject for age groups is shown in Fig. 3(b)-right. The $\Delta Pa_{800}$ increased with age, i.e., 1.8, 2.2, and 2.7 dB for the age group of subjects in their 20s, 30s, and 40s, respectively.

**Table 2:** The values of minimum ($Pa_{\min}$) and maximum ($Pa_{\max}$) of PA power and the difference between $Pa_{\min}$ and $Pa_{\min}$ (ΔPa) at 700, 800, and 900 nm for all groups. The error bars represent the standard deviation from the number of cycles of the pulsatile flow for all subjects.

Group	20s			30s			40s		
λ (nm)	700	800	900	700	800	900	700	800	900
$Pa_{\max}$ (dB)	−4.8±0.7	1.8±0.3	7.0±0.5	−3.8±0.6	2.2±0.4	7.3±0.6	−3.0±0.5	2.7±0.3	7.9±0.4
$Pa_{\min}$ (dB)	−6.3±0.6	0	4.8±0.5	−5.6±0.6	0	4.4±0.6	−5.2±0.5	0	4.2±0.5
ΔPa (dB)	1.5±0.2	1.8±0.3	2.2±0.4	1.8±0.2	2.2±0.4	2.9±0.4	2.2±0.2	2.7±0.3	3.7±0.4

### 3.2 sO_2_ versus Subject Age

**Fig. 4:**
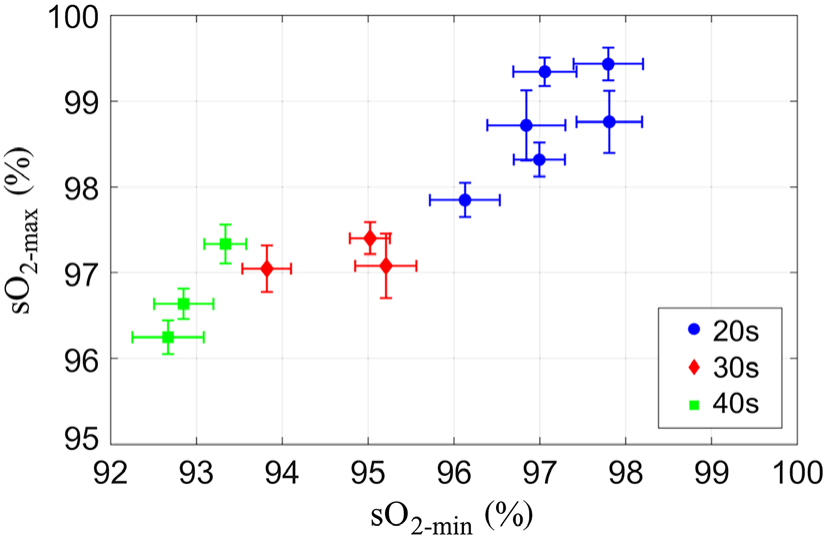
Oxygen saturation (${\mathrm{sO}}_{2}$) for all subjects—subjects in their 20s (blue dots), subjects in their 30s (red diamonds), and subjects in their 40s (green square). The ${\mathrm{sO}}_{2}$ was computed for $Pa_{\max}$ (${\mathrm{sO}}_{2\text{-}\max}$) and $Pa_{\min}$ (${\mathrm{sO}}_{2\text{-}\min}$), respectively. The error bars represent the standard deviation from the number of cycles of the pulsatile flow.

**Fig. 5:**
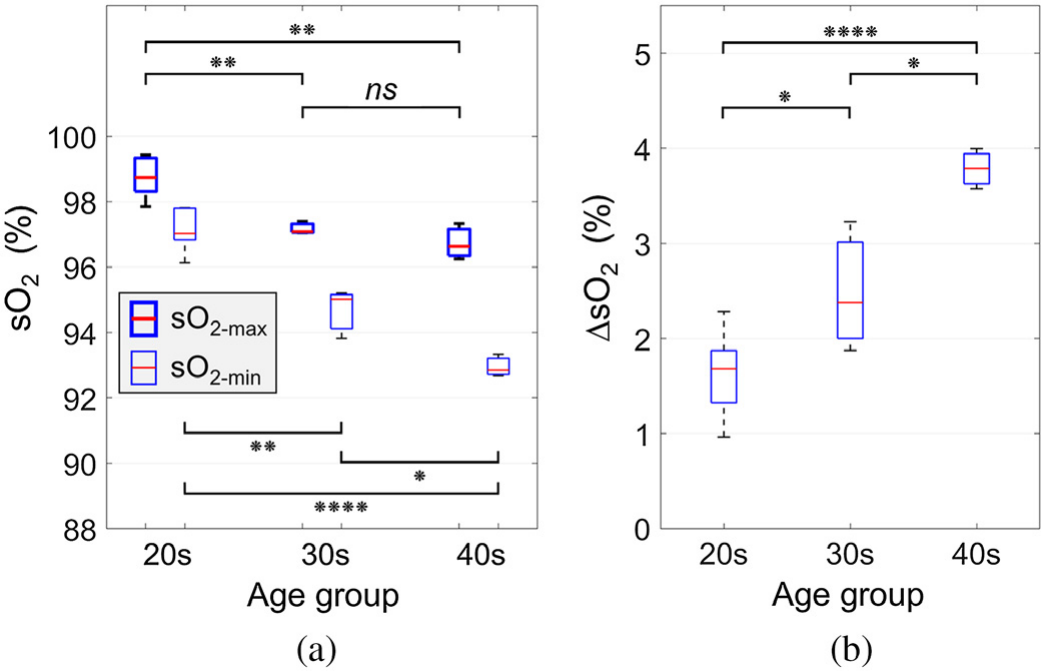
(a) Oxygen saturation (${\mathrm{sO}}_{2}$) for each group based on the average value of ${\mathrm{sO}}_{2}$ of each subject. The thick (${\mathrm{sO}}_{2\text{-}\max}$) and thin (${\mathrm{sO}}_{2\text{-}\min}$) symbols represent the ${\mathrm{sO}}_{2}$ computed using the two wavelength (700 and 900 nm) methods at $Pa_{\max}$ and $Pa_{\min}$, respectively. (b) The difference between ${\mathrm{sO}}_{2\text{-}\max}$ and ${\mathrm{sO}}_{2\text{-}\min}$ ($\Delta {\mathrm{sO}}_{2}$) for each group. ns: not signigicant, *: p≤0.05, **: p≤0.01, ****: p≤0.0001.

The ${\mathrm{sO}}_{2}$ estimated from $Pa_{\min}$ (${\mathrm{sO}}_{2\text{-}\min}$) and $Pa_{\max}$ (${\mathrm{sO}}_{2\text{-}\max}$) for each group of age 20s, 30s, and 40s were shown in Fig. 4, respectively. The average ${\mathrm{sO}}_{2\text{-}\max}$ for subjects in their 20s, 30s, and 40s were 98.7%, 97.2%, and 96.7%, respectively [Fig. 5(a)]. This indicates that the difference in ${\mathrm{sO}}_{2\text{-}\max}$ between the youngest and oldest subjects was 2.0% (p≤0.01). On the other hand, the average ${\mathrm{sO}}_{2\text{-}\min}$ for subjects in their 20s, 30s, and 40s were 97.1%, 94.7%, and 93.0%, respectively [Fig. 5(a)]. The ${\mathrm{sO}}_{2\text{-}\min}$ difference between the 20s and 40s groups was 4.1% (p≤0.0001). The ${\mathrm{sO}}_{2}$ difference between ${\mathrm{sO}}_{2\text{-}\min}$ and ${\mathrm{sO}}_{2\text{-}\max}$ ($\Delta {\mathrm{sO}}_{2}$) during a pulsatile cycle increased with age, i.e., 1.6%, 2.5%, and 3.8% for the age group of subjects in their 20s, 30s, and 40s, respectively [Fig. 5(b)].

## 4 Discussion

Several studies have demonstrated the PA assessment of the ${\mathrm{sO}}_{2}$.^28^^,^^46^^,^^49^^,^^50^ However, the complexity of hemodynamic and/or hemorheological behaviors such as RBC aggregation/disaggregation and deformation have not been considered as a possible factor that could alter the estimated ${\mathrm{sO}}_{2}$. Our group has studied the feasibility of using PA spectroscopy for assessing the relation between RBC aggregation and ${\mathrm{sO}}_{2}$ under pulsatile blood flow *in vitro* using experimental and theoretical methods.^41^^,^^42^ This paper describes the first-in-human feasibility study on the dependence of the ${\mathrm{sO}}_{2}$ on the pulsatile blood flow conditions of the radial artery. The experimental results show an age dependence of the ${\mathrm{sO}}_{2}$ measurements *in vivo*.

### 4.1 PA Power versus Age – Aggregability of RBCs

Several studies reported on the correlation between RBC aggregation and age.^51^^–^^53^ Woodward et al.^51^ measured hemorheological variables (blood viscosity, RBC aggregation, and fibrinogen) from subjects (25 to 74 years old) in association with cardiovascular risk factors. They found that RBC aggregation and fibrinogen increased with age. Christy et al.^52^ reported a significant increase in RBC aggregation with age (20 to 59 years old), establishing a significant correlation between phagocytic activity and RBC aggregability. According to Simmonds et al.^53^ the mechanism of the age-related increase in RBC aggregation is that aging decreased the electrostatic repulsive forces between cell surfaces, thus promoting RBC aggregation alongside with increased plasma fibrinogen concentration.

During pulsatile blood flow, the dominant hemorheological characteristic is the cyclical aggregation and disaggregation phases of RBCs.^5^^,^^32^ During systole, the blood flow velocity is maximum, generating maximal shear rate within vessels, and as a result, the RBCs in the radial artery flow as single cells. During diastole, on the other hand, the blood flow velocity is at a minimum, resulting in a minimal shear rate, leading to the formation of RBCs rouleaux.^5^^,^^32^ In PA imaging, the RBC aggregates form a larger effective absorber compared to single cells, increasing the Pa signal.^41^^,^^42^^,^^54^^,^^55^ In Fig. 2(b), the Pa and Doppler velocity V were out of phase during a systolic-diastolic cycle. A higher Pa is expected in the presence of aggregates and a lower Pa from single RBCs. This occurred at all wavelengths of illumination, and as we have shown in earlier studies, it can approximate the aggregate size.^41^

The PA amplitude is a function of $\mu_{a}$ (including the absorption cross-section) and an absorber size. According to Eq. (1), $\mu_{a}$ can be represented as a linear combination of $\varepsilon_{\mathrm{HbO}}$, $\varepsilon_{\mathrm{HbD}}$, [HbO] and [HbD], depending on λ. The ${\mathrm{sO}}_{2}$ can be derived by combining $\mu_{a}$ at two wavelengths as addressed in Eq. (2). Given the ${\mathrm{sO}}_{2}$ also varies with the absorber size affecting the $\mu_{a}$, the Pa must have a nonlinear relation with the absorber size. However, the Pa at an isosbestic point 800 nm is not dependent on the ${\mathrm{sO}}_{2}$, resulting in a linear relation between the Pa and the absorber size, as previously demonstrated by our group.^41^ The $\Delta Pa_{800}$ increased with age [Fig. 3(b)], suggesting that the RBC aggregability also increased with age (since the PA signal is a surrogate metric of the aggregate size).

### 4.2 Effect of RBC Aggregation on PA Power as a Function of Absorber Size and sO_2_

None of the abovementioned studies have examined the impact of RBC aggregation on the blood ${\mathrm{sO}}_{2}$ measurements in humans. It is well known that RBC aggregation and plasma viscosity play an important role in determining the overall blood viscosity.^56^ Realizing the fundamental function of RBCs in oxygen transport, Tateishi et al.^57^^,^^58^ were the first to postulate on the correlation between RBC aggregation and $O_{2}$ release from erythrocytes and its impact on the ${\mathrm{sO}}_{2}$.^59^ In addition, the relation between RBC aggregation and the ${\mathrm{sO}}_{2}$ was theoretically and experimentally investigated through *in vitro* PA imaging by our group.^41^ Similar to the Tateishi’s findings and our *in vitro* experiments, the *in vivo* results in this work demonstrate the impact of RBC aggregation on the ${\mathrm{sO}}_{2}$ measurement.

**Fig. 6:**
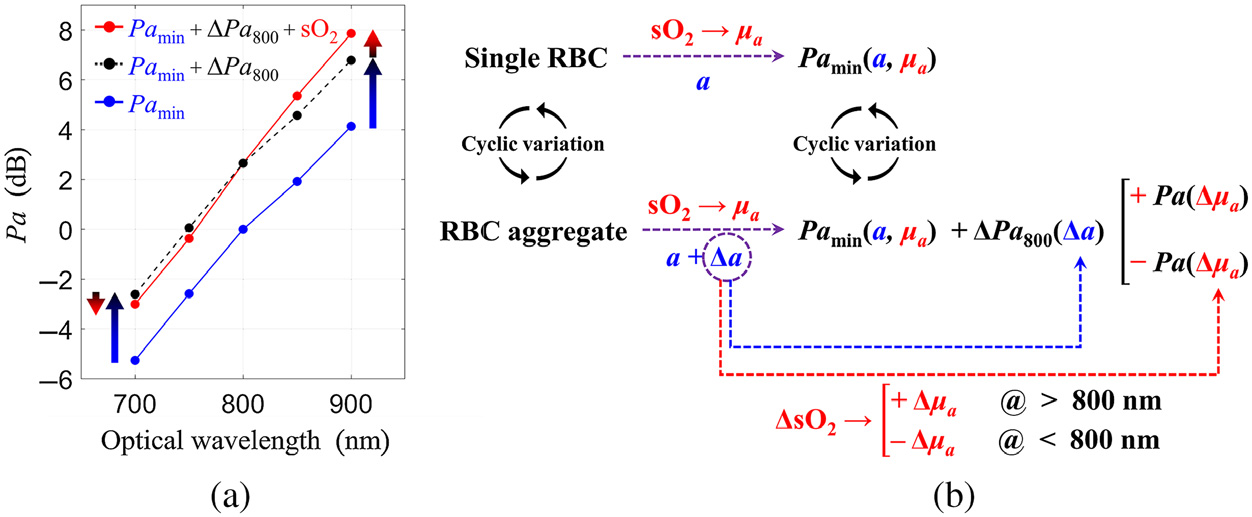
(a) The effect of RBC aggregation on PA power (Pa) in relation to an optical absorber size and oxygen saturation (${\mathrm{sO}}_{2}$). (b) Schematic diagram of the effect of RBC aggregation on Pa in relation to the absorber size and the ${\mathrm{sO}}_{2}$. a and Δa represent the size of single RBC and the increased size due to aggregation, respectively.

The Pa increases due to two factors: an increase in the size of the absorber (single cells versus RBC aggregates) and the change in ${\mathrm{sO}}_{2}$. The average values of Pa as a function of λ for the group of subjects in their 40s are shown in Fig. 6(a). The $Pa_{\min}$ represents measurements when nonaggregated RBCs flow in the radial artery at maximal velocity (when the shear rates inside the radial artery are highest). RBC aggregation increases the Pa due to an increase in optical absorber size. Since the optical absorber size increases as RBCs aggregate, the $Pa_{\min}$ increases by $\Delta Pa_{800}$ (2.7 dB for subjects in their 40s) for all λ ($Pa_{\min}+\Delta Pa_{800}$, depicted by the blue-black arrow). This can be schematically represented by $\Delta Pa_{800}(\Delta a)$ in Fig. 6(b). The second reason for the changes in the Pa is the change in the ${\mathrm{sO}}_{2}$ due to RBC aggregation. The difference between $Pa_{\max}$ and “$Pa_{\min}+\Delta Pa_{800}$” can be identified as the contribution to the change in ${\mathrm{sO}}_{2}$ caused by RBC aggregation to the overall PA signal, ($Pa_{\min}+\Delta Pa_{800}+{\mathrm{sO}}_{2}=Pa_{\max}$). Combining Eqs. (1) and (2), the $\mu_{a}$ can be expressed as a function of ${\mathrm{sO}}_{2}$,

$$\mu_{a}=((\varepsilon_{\mathrm{HbO}}-\varepsilon_{\mathrm{HbD}}){\mathrm{sO}}_{2}+\varepsilon_{\mathrm{HbD}})([\mathrm{HbO}]+[\mathrm{HbD}]).$$ (4)

Since $\varepsilon_{\mathrm{HbO}}$ is larger than $\varepsilon_{\mathrm{HbD}}$ for λ>800  nm,^60^ the ${\mathrm{sO}}_{2}$-induced increase in $\mu_{a}$ results in an increased Pa with ${\mathrm{sO}}_{2}$, as shown in Fig. 6. On the other hand, since $\varepsilon_{\mathrm{HbO}}$ is smaller than $\varepsilon_{\mathrm{HbD}}$ for λ<800  nm, the ${\mathrm{sO}}_{2}$-induced decrease in $\mu_{a}$ results in a decreased Pa. This can be schematically denoted by $+Pa(\Delta \mu_{a})$ and $-Pa(\Delta \mu_{a})$ for λ>800  nm and λ<800  nm, respectively, in Fig. 6(b). The *in vivo* experimental results demonstrating the effect of RBC aggregation on the Pa and how this depends on the RBC ${\mathrm{sO}}_{2}$ are also supported by our group’s previous *in vitro* work mimicking the radial artery flow conditions.^41^ This interpretation applies to all age groups.

### 4.3 Relationship between sO_2_ versus Age

$Pa_{\max}$ could be derived from $Pa_{\min}$ by combining the effect of the increase in both absorber size and ${\mathrm{sO}}_{2}$ due to RBC aggregation. Hence, the ${\mathrm{sO}}_{2}$ computed from the $Pa_{\max}$ (${\mathrm{sO}}_{2\text{-}\max}$) were higher than that from the $Pa_{\min}$ (${\mathrm{sO}}_{2\text{-}\min}$) for all groups [as shown in Fig. 5(a)]. In addition, both ${\mathrm{sO}}_{2\text{-}\max}$ and ${\mathrm{sO}}_{2\text{-}\min}$ decreased with increasing age. It has been reported that older persons have a lower ${\mathrm{sO}}_{2}$ than their younger counterparts.^61^^–^^65^ Even though the direct relation between the ${\mathrm{sO}}_{2}$ and age has not been fully investigated, it was reported that dysphagia (swallowing difficulties) could mediate the relation between ${\mathrm{sO}}_{2}$ and age.^65^ Older people are more likely to experience dysphagia which impairs pulmonary function and lowers ${\mathrm{sO}}_{2}$. The present experimental results indicated that the ${\mathrm{sO}}_{2}$ decreases with age, despite the subject cohort comprising healthy individuals.

The difference between ${\mathrm{sO}}_{2\text{-}\max}$ and ${\mathrm{sO}}_{2\text{-}\min}$ ($\Delta {\mathrm{sO}}_{2}$) increased with age, as shown in Fig. 5(b). This phenomenon was consistent with the relation between $\Delta Pa_{800}$ and age, as shown in Fig. 3(b). The $Pa_{800}$ represents the effects of RBC aggregability, independent of ${\mathrm{sO}}_{2}$, which increased with age. Since oxygen release is inhibited by RBC aggregation,^57^^,^^58^ higher aggregability results in more hemoglobin molecules to be bound oxygen molecules. Despite a negative correlation between the ${\mathrm{sO}}_{2\text{-}\min}$ and age, the RBC aggregation-induced increase in the ${\mathrm{sO}}_{2}$ ($\Delta {\mathrm{sO}}_{2}$) resulted in a positive correlation between $\Delta {\mathrm{sO}}_{2}$ and age. This is why the correlation between the ${\mathrm{sO}}_{2\text{-}\min}$ and age is steeper than the correlaiton between the ${\mathrm{sO}}_{2\text{-}\max}$ and age, as shown in Fig. 5(a).

### 4.4 Limitations of the Study

The findings of this study suggest the feasibility of the PA assessment of both ${\mathrm{sO}}_{2}$ and its correlation to age, *in vivo*. However, there are limitations to this study that form the basis for future extension of this work. The study, whose recruitment is suspended during the global COVID-19 pandemic, enrolled a smaller than desired subject group and was mainly composed of male volunteers. The sex dependence on Pa related to the correlation between ${\mathrm{sO}}_{2}$ and age must be further investigated. The laser fluctuation which sometimes occur from pulse to pulse or from wavelength to wavelength should be measured and corrected. The absolute values for the ${\mathrm{sO}}_{2}$ presented in this paper have not been corrected for the effects of laser fluence.^66^ The radial artery depth from the skin surface is ∼5  mm. As such, the effects of fluence (spectral coloring)^67^ might not be as significant as it is in other PA applications. Specifically, in Eq. (3), the ${\mathrm{sO}}_{2}$ is calculated by a ratio of $\mu_{a}$. The PA amplitude (P) is a function of $\mu_{a}$ and fluence (φ), so that the ${\mathrm{sO}}_{2}$ can be represented by the ratio of “P/φ.” Moreover, since the position and geometry were the same, variations in fluence are minimized. In addition, the normalization to the laser energy at each wavelength could also contribute to a reduction of the fluence effects. As such, the reported absolute values might not represent the true ${\mathrm{sO}}_{2}$ for each subject. In addition, this quantity is dependent on the subject’s skin color (leading to a larger optical path length to the radial artery, requiring fluence correction). An additional limitation is that the upper limit of age group was in 40s. Further investigation for the older age groups (50s, 60, and 70s) should be conducted to study the age dependence further.

Despite these limitations, this study demonstrates the feasibility of the measurements and confirms previous *in vitro* findings. Future studies could be done using a portable probe, opening the potential for doing this investigation more easily. In addition, quantitative measurement of RBC aggregation by US such as the structure-factor-size-estimation^68^ should be further applied to this study to correlate both PA and US modalities.

## 5 Conclusion

In this study, the correlation between the ${\mathrm{sO}}_{2}$ and RBC aggregation under the pulsatile blood flow in the human radial artery, and its age dependence, was investigated using PA imaging. RBC aggregability increased with age, as observed by the age-induced increase in the $\Delta Pa_{800}$ metric during RBC aggregation. The ${\mathrm{sO}}_{2}$ change during a cycle of pulsatile blood flow also increased with age, and it was attributed to the presence of increased RBC aggregation in older subjects. This study is the first study to examine how the *in vivo* changes in ${\mathrm{sO}}_{2}$ during blood flow in human body can be assessed using PA imaging. This study demonstrates the effect of RBC aggregation on the ${\mathrm{sO}}_{2}$ change during a cardiac cycle in healthy volunteers and the increase in the ${\mathrm{sO}}_{2}$ change with age. Although these preliminary observations were conducted in only 12 healthy participants, this work demonstrates the feasibility of the measurement *in vivo*. Such measurements might shed light on the clinical importance of the complex relation between the blood viscosity induced by RBC aggregation and the oxygen delivery related to ${\mathrm{sO}}_{2}$. As a noninvasive measurement, PA imaging of blood ${\mathrm{sO}}_{2}$ could be extended to the detection of blood pathologies that alter the viscosity, modifying flow behaviors and oxygen delivery.
